# Terrestrial records of deglaciation events during terminations V and IV in the central Apennines (Italy) and insights on deglacial mechanisms

**DOI:** 10.1038/s41598-022-23391-7

**Published:** 2022-11-05

**Authors:** F. Marra, A. Pereira, B. Jicha, S. Nomade, I. Biddittu, F. Florindo, G. Muttoni, E. M. Niespolo, P. R. Renne, V. Scao

**Affiliations:** 1 Istituto Nazionale di Geofisica e Vulcanologia, Rome, Italy; 2 Université Paris-Saclay, CNRS UMR 8148, GEOPS, Gif-sur-Yvette, France; 3 Département Hommes et Environnements, Muséum National d’Histoire Naturelle, Paris, France; 4 Department of Geoscience, University of Wisconsin-Madison, Madison, USA; 5 CEA Saclay, LSCE, UMR-8212, UVSQ-IPSL et Université Paris Saclay, Gif-Sur-Yvette Cedex, France; 6 Istituto Italiano di Paleontologia Umana, Anagni, Italy; 7 Department of Earth Sciences, University of Milan, Milan, Italy; 8 Department of Earth and Planetary Science, University of California, Berkeley, USA; 9 Berkeley Geochronology Center, Berkeley, USA; 10 Department of Geosciences, Princeton University, Princeton, USA

**Keywords:** Palaeoclimate, Stratigraphy

## Abstract

^40^Ar/^39^Ar geochronology constraints to aggradational phases and grain size variations show that the two large gravel beds occurring in the sedimentary filling of the Liri fluvial-lacustrine basin (central Italy) recorded the occurrence of deglaciation events synchronous within uncertainties with global meltwater pulses at ca. 450 and 350 ka. In particular, we find a precise match between the ages of gravel deposition and the occurrence of moderate sea-level rise events which anticipate those more marked during the glacial termination V and IV in the Red Sea relative sea level curve, as already verified by data from the Tiber River catchment basin. Such correspondence suggests that gravel deposition is facilitated by melting of Apennine mountain range glaciers, which provide the water transport energy and a surplus of clastic input to the rivers draining the mountain regions and flowing into the Tyrrhenian Sea. Therefore, the thick gravel beds intercalated in the sedimentary filling of the catchment basins of the major rivers in central Italy may be regarded as an equivalent proxy of large deglaciation events, similar to the ice-rafted debris in northern Atlantic. Consistent with this hypothesis, we also show the close correspondence between the occurrence of particularly mild (warmer) minima of the mean summer insolation at 65° N and these early aggradational phases, as well as with other anomalous early sea-level rises occurring c. 750 ka and 540 ka at the onset of glacial termination VIII and VI, and 40 ka at the onset of the so-called Heinrich events.

## Introduction

Geochronologically constrained records of glacial–interglacial variations in ice volume and sea level represent a fundamental tool to decipher Pleistocene global climate evolution. However, records characterized by global rather than local significance are very rare (e.g., ocean cores, ice cores, coral reefs, speleothems) and those with direct, precise radioisotopic age constraints, especially in the time interval > 500 ka, are even less common.

**Figure 1:**
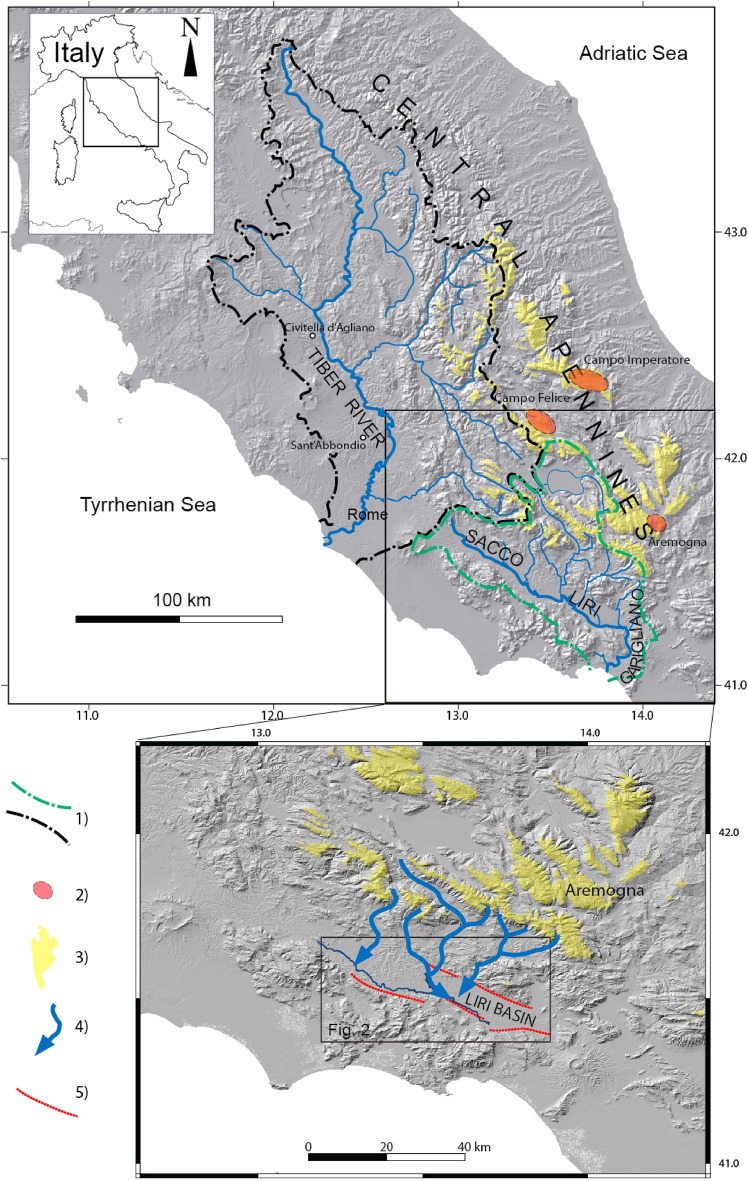
1) Catchment basins of the Tiber River (black border) and of the Sacco-Liri-Garigliano Rivers (green border); 2) areas with evidence of glacial landforms associated with MIS 14 through MIS 6 glaciations and (3) areas above 1500 m a.s.l. of potential expansion of the glaciers according to Giraudi and Giaccio^16^; 4) meltwater and clastic inputs reaching the Liri Basin during deglacial periods; 5) main border faults of the Sacco-Liri basins. The location of the investigated area represented in Fig. 2 is shown. DEM images: TINITALY/01 square WA 6570, used with permission of the Istituto Nazionale di Geofisica e Vulcanologia, Rome. Graphics handrawn by authors.

Over 30 years of dedicated studies have shown that fluvial-lacustrine sedimentation within the catchment basin of the Tiber River (Fig. 1) responded synchronously with changes in base-level induced by glacio-eustatic fluctuations during Middle-Upper Pleistocene^1–15^.

The sedimentary record of the Paleo-Tiber River consists of a series of fining-upward sequences of clastic sediments deposited above an erosional surface in response to sea-level rise during the last eight glacial–interglacial transitions (‘aggradational successions’)^17^ (and references therein). Each sequence is characterized by a basal interval of distinct gravel deposition that abruptly switches to clay deposits. In particular, ^40^Ar/^39^Ar age constraints on tephra layers interbedded with the sedimentary deposits indicate that coarse gravel beds at the base of each aggradational succession are deposited as a consequence of ice-melting during the glacial termination. These results have highlighted that conditions for the accumulation of the coarse gravel deposits only coexisted at the onset of glacial terminations due to several concurrent factors:

1. low sea level at glacial maxima, which steepens the gradients and, in turn, enhances the river competence through the more deeply incised valley;
2. melting of Apennine mountain chain glaciers that releases large amounts of clastic material, increasing the sediment supply to the river drainage basin;
3. overall increase in regional precipitation.

Gravel starts accumulating at the end of the glacial period, when sea-level continues to fall, which caused re-incision of the valley floor and removal of the gravel transported during the regressive phase up to the glacial maximum. The abruptness of the “sedimentary switch” that marks the transition from the gravel bed (2–8 m in thickness), through a thin (< 1 m in thickness) sand bed, into the thick (20–40 m in thickness) silt and clay section calls for the sudden establishment of a low gradient, consistent with fast sea-level rise (meltwater pulse) and subsequent development of a sea-level highstand.

Previous work focused on the aggradational successions in the coastal plain and the terminal tract of the Tiber River. More recent studies have highlighted the synchronicity, within uncertainties of age models, between the gravel/clay switch and the peaks of sea-level rise during Marine Isotopic Stage (MIS) 11 and MIS 9 in the higher portion of the Tiber catchment basin, as far inland as 50 km^14^ and 100 km^9^ from the coast (Sant'Abbondio and Civitella di Agliano locations in Fig. 1).

Giaccio et al.^14^ have remarked on the coincidence among melt-water pulse events, peaks in the ice-rifted debris (IRD) curve^18^, and deposition of the gravel beds of the MIS 11 aggradational succession (San Paolo Formation^2^), suggesting that gravel deposition in the catchment basins of the Tiber River can be regarded as an equivalent proxy of deglaciation events. This composite record of radioisotopically dated (^14^C and ^40^Ar/^39^Ar) morpho-sedimentary units can thus provide key geochronological constraints that are generally lacking in the Middle Pleistocene sea-level records, and they can be used to better evaluate the relationship between insolation changes and sea-level oscillations.

In order to verify the global significance of this kind of proxy and the possibility to use similar morpho-stratigraphic units from other regions to constrain the timing of the deglacial phases, we have investigated the possible response to the glacio-eustatic signal of sediment supply grainsize within the Sacco-Liri-Garigliano Rivers catchment basin, located in central-southern Italy (Fig. 1).

Here, a more than 50 m thick fluvial-lacustrine succession filled the tectonic depression of the Liri basin during the Middle Pleistocene^19–21^.

**Figure 2:**
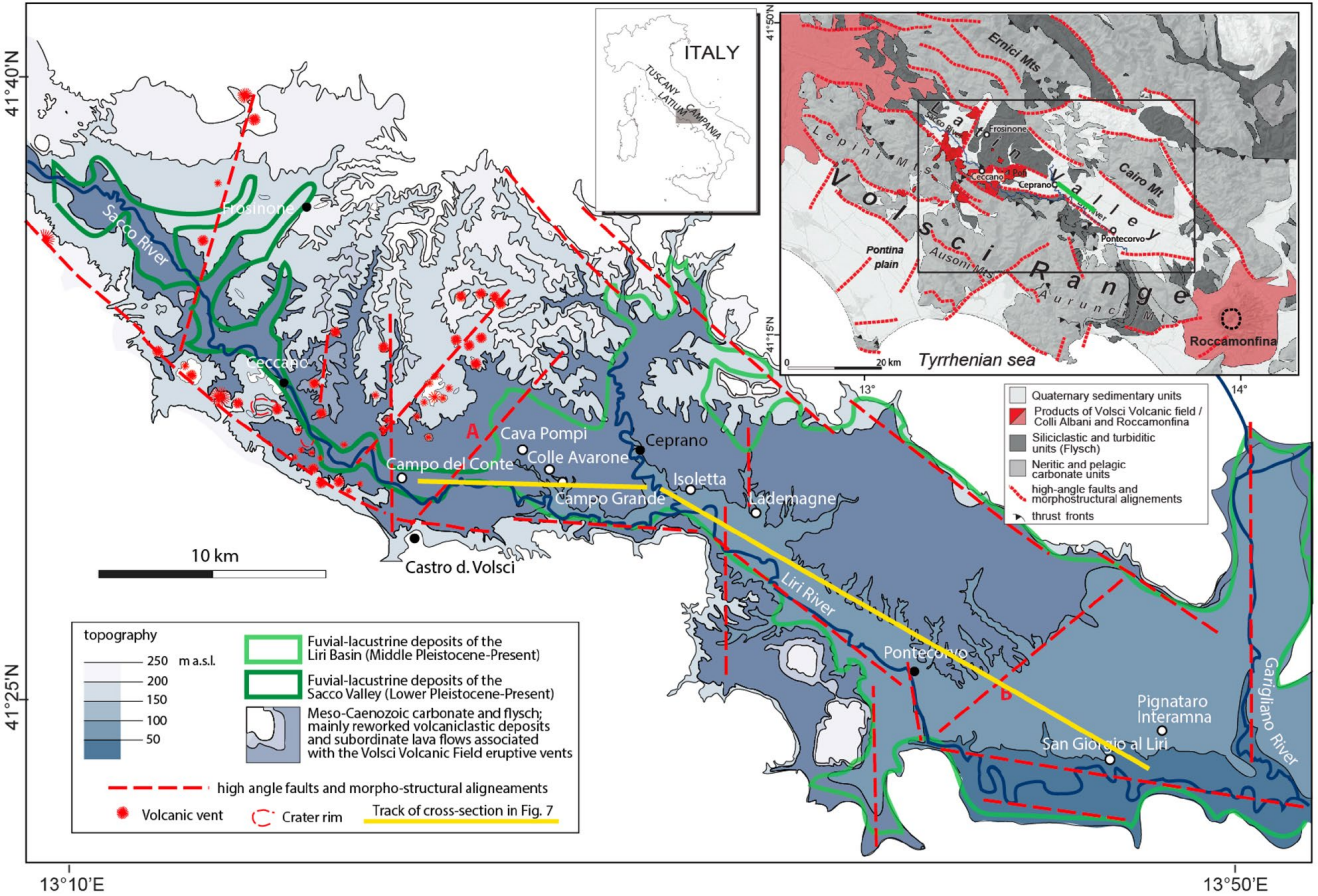
(**a**) Morpho-structural sketch of the Latin Valley with the Sacco-Liri catchment basin showing location of the investigated geologic sections (white dots). (**b**) Regional geologic map (created by authors) straddling the investigated area. All graphics handrawn by authors.

In this study, we identify two 3–4 m thick beds of coarse gravel deposits intercalated within clayey lacustrine sediment of the Middle-Pleistocene Sacco-Liri basin (Fig. 2). We correlate these gravel beds cropping out at three geologic sections (Colle Avarone, San Giorgio al Liri, Pignataro Interamna) and re-analyze the stratigraphy of six sites described in the literature (Campo del Conte, Cava Pompi, Campogrande, Isoletta, Lademagne, Pontecorvo) located in three different structural portions of the Sacco-Liri basin (Fig. 2). Detailed stratigraphic sketches of these sections are reported in Suppl. Mat. #1.

In order to provide age constraints to the time of deposition of these gravel beds, we produced 8 new ^40^Ar/^39^Ar ages both on primary volcanic samples and reworked deposits containing potassium (K)-feldspar crystals, which we integrated with 9 ^40^Ar/^39^Ar and 2 K/Ar dates previously produced from these sections.

A detailed description of the sedimentary features of the Sacco-Liri basin can be found in Devoto^19^. This author described the sedimentary succession filling the large lacustrine basin in the "Lower Liri Valley", between Ceprano and the Garigliano River confluence (Fig. 2), as composed of three lacustrine facies with vertical and lateral transition from one to another:

1. *Lower lacustrine mud*. Bedded white calcareous muds with frequent intercalated black tephra.
2. *Typical lacustrine facies*. White calcareous muds with alternating cross-bedded yellow sand layers, black and brown "tuffite" with slumpings, conglomerate.
3. *Late lacustrine facies*. Calcareous muds varying in color with occasional lignite and peat layers, transitioning laterally and vertically to travertine.

The occurrence of conglomerate deposits is only briefly mentioned by Devoto^19^ in the description of the "Typical lacustrine facies". Such high energy sediments within a large lacustrine basin may have a differing origin, possibly related to e.g., a local alluvial fan or sedimentary traps. However, our study evidenced that coarse gravel layers are concentrated within two horizons occurring at different elevation. The upper one, 2–3 m thick, can be laterally correlated for several kilometers through the investigated sections. The lower gravel bed, ca. 4 m thick, has been found in a borehole at Ceprano^22^, where it occurs at the base of a ca. 20 m thick package of lacustrine muds of the "Typical lacustrine succession", and is reported by Devoto^19^ to occur in the south-eastern portion of the basin within this sedimentary unit.

Such gravel occurrences are not repeated in the upper, younger levels of the Liri basin succession, suggesting that its emplacement is linked with significant variation in water capacity of transport that affected in two distinct periods of the past the Sacco-Liri catchment basin. We interpret these to correlate with melting of the glaciers occurring in the high ranges of the central Apennines. The presence of glaciers in the central Apennines during past glacial periods has been shown by a review of the existing data for the Middle Pleistocene glacial remnants and new stratigraphic and tephrochronological data which enabled Giraudi and Giaccio^16^ to recognize at least five glacial stages during MIS 14, 12, 10, 8 and 6. In particular, one of these areas is located at the northern margin of the Sacco-Liri-Garigliano catchment basin (Aremogna, Fig. 1). Glacial landforms associated with the Apennine's glaciers have been reported to occur at elevations as low as 1500 m a. s. l.^16^ (and references therein).

### Geological-structural setting

The Latin Valley (Fig. 2) is part of the central Apennine fold-and-thrust belt, which originated from Late Tortonian-Early Messinian compressional phases and has been affected by extensional tectonics since the Pliocene^23,24^. The outcropping terrains belong to the Latium-Abruzzi neritic carbonate domain (upper Triassic-middle Miocene) and are covered by middle Miocene to lower Pliocene syn-orogenic siliciclastic deposits^25^.

The study area is organized in several NW–SE striking imbricate thrust sheets that overthrust onto Tortonian-lower Messinian terrigenous deposits, cross-cut by a system of conjugated synthetic and antithetic Quaternary normal faults which controlled the formation and growth of intramountain basins during the extensional phase^26^. The articulated catchment basin of the Sacco, Liri and Garigliano Rivers (Figs. 1, 2) developed in a graben structure within the Latin Valley^26^.

Locally, the study area has also been affected by N- to NNE- and E- to ENE-striking high angle faults with strike-slip kinematics up to the Middle Pleistocene^21,26,27^. The strike-slip tectonics were associated with the eruptive centers of the Volsci Volcanic Field^28^, the activity of which occurred in three main phases^21^. An early eruptive phase spanning 761.5 ± 9.5 to 541.0 ± 14.0 ka (all errors 2σ) was characterized by long quiescent periods between isolated eruptive events; major eruptive activity occurred from 424.0 ± 13.0 to 349.5 ± 5.0 ka; finally, a late eruptive phase has less precise geochronologic constraints between 300.0 ± 28.0 and 231.0 ± 19.0 ka^21^.

## Results

### ^40^Ar/^39^Ar data

**Figure 3:**
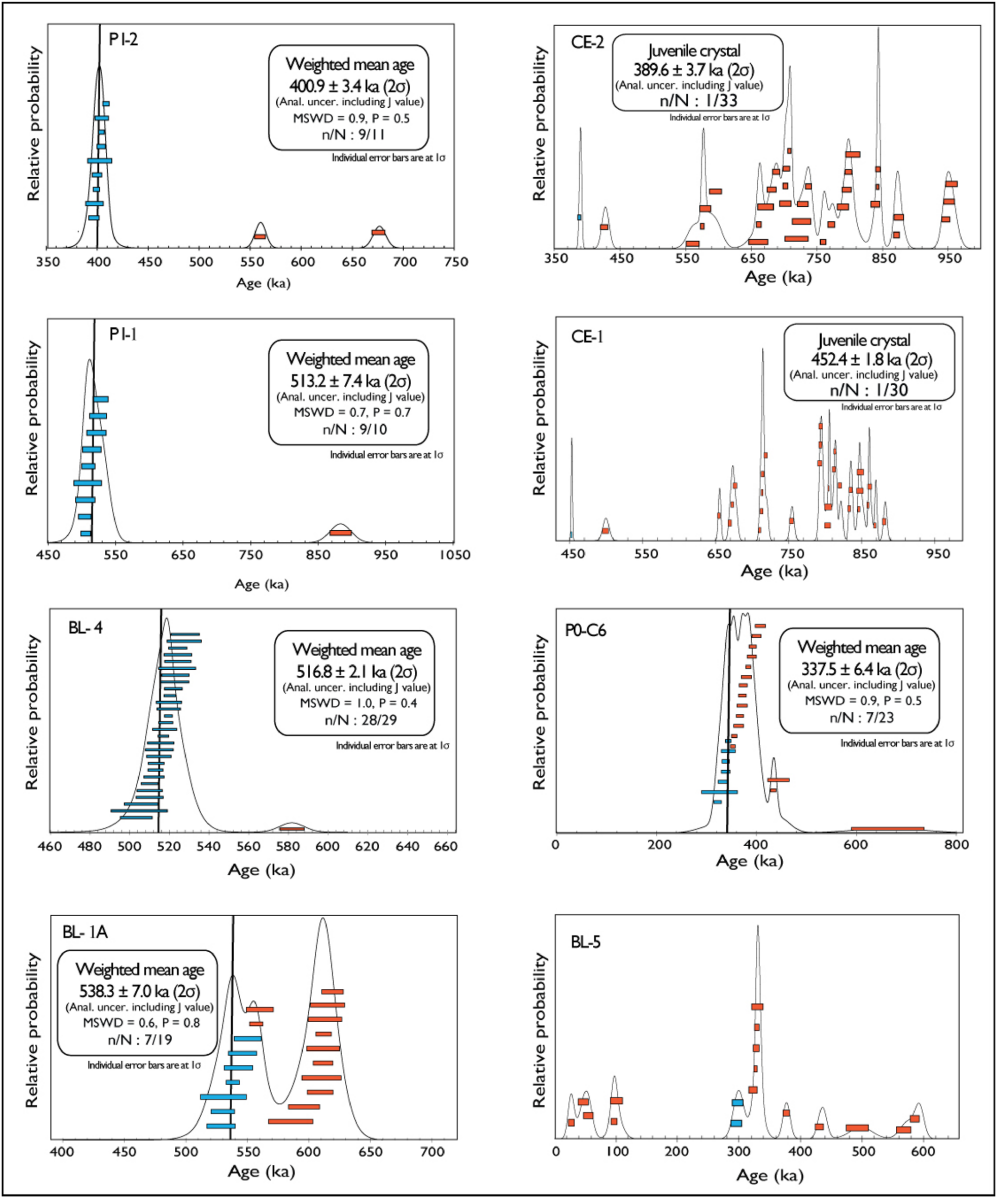
Results of ^40^Ar/^39^Ar analyses on single crystals from this study presented as probability diagrams.

Results for the eight samples dated in the present study are reported, along with those of 9 previous samples, as age probability diagrams in Fig. 3 and summarized in Table 1. Full analytical data are reported in Supplementary Material #2.

**Table 1:** ^40^Ar/^39^Ar sample ages. All ages according to ACs at 1.1848 Ma^29,30^.

Sample/site	Age (ka) ± 2σ	n/N	MSWD	Latitude Longitude	Elev (m asl)
BL-5	300 ± 12	2/17	–	41° 22′ 22.16″ N 13° 45′ 58.36″ E	48.5
PO-C6	337.5 ± 6.4	7/23	0.90	41° 33′ 31.24″ N 13° 26′ 18.58″ E	129.5
CE-2	389.6 ± 3.7	1/33	–	41° 31′ 43.35″ N 13° 28′ 45.92″ E	95
PI-2	400.9 ± 3.4	9/11	0.90	41° 26′ 05.37″ N 13° 47′ 30.12″ E	50
PI-1	513.2 ± 7.4	9/10	0.70	41° 26′ 05.37″ N 13° 47′ 30.12″ E	48
BL-4	516.8 ± 2.1	28/29	1.04	41° 22′ 22.16″ N 13° 45′ 58.36″ E	47
BL-1A	538.3 ± 7.0	7/19	0.56	41° 22′ 22.16″ N 13° 45′ 58.36″ E	40.5
CE-1	452.4 ± 1.8	1/30	–	41° 31′ 43.35″ N 13° 28′ 45.92″ E	72
**From previous literature**					
CA-C1^21^	345.9 ± 4.3	8/28	0.82	41° 32′ 11.80″ N 13° 28′ 41.40″E	119.5
Ceprano^33^	350.6 ± 8.0	14/17	1.50		
CA-CGT^21^	359.6 ± 6.5	3/24	0.04	41° 32′ 11.80″ N 13° 28′ 41.40″ E	112.5
Isoletta 3^35^	362.6 ± 3.8	2/8	0.34	41° 31′ 44.86″ N 13° 34′ 0.16″ E	112
Lademagne 2^35^	386.2 ± 4.6	7/17	1.60	41° 31′ 16.68″ N 13° 35′ 0.72″ E	105
Isoletta 2^35^	373.2 ± 2.8	10/13	1.40	41° 31′ 44.86″ N 13° 34′ 0.16″ E	104
Cava Pompi^35^	394.3 ± 8.2	8/8	0.25	41° 33′ 31.24″ N 13°26′ 18.58″ E	127
Isoletta 1^35^	400.3 ± 3.0	11/12	0.85	41° 31′ 44.86″ N 13° 34′ 0.16″ E	92
Lademagne 1^31^	402.4 ± 4.8	7/15	1.10	41° 31′ 16.68″ N 13° 35′ 0.72″ E	101.8

### Chronostratigraphic analysis of the Sacco-Liri basin

#### Campo del Conte

A sedimentary sequence about 7 m in thickness, including four fluvial depositional cycles, crops out in the western sector of the Latina Valley in Campo del Conte^31^ (Fig. 2). This sequence is characterized by the occurrence of *Mammuthus meridionalis* fossils along with those of a cervid belonging to the Pseudo-dama group^31^ indicating an Early Pleistocene age. More specifically, *M. meridionalis* lived in central Italy during the period chronologically framed between 2.6 and 1.6 Ma^32^.

#### Campo Grande (Ceprano boreholes)

Two boreholes drilled in the western sector of the Liri basin in Campo Grande (Fig. 2) recovered 48 m of lacustrine succession between 108 and 60 m a.s.l., without reaching its bottom^22^. Three main successions, the lowest two separated by a ca. 5 m thick gravel layer, were tentatively correlated with the three lacustrine facies described by Devoto^19^. Although not strictly applicable everywhere, we will use these three successions, here termed "lower lacustrine", "middle lacustrine-fluvial", and "upper fluvial-lacustrine", as reference chronostratigraphic units in this paper.

We have collected two samples from the Campo Grande borehole cores stored at Università degli Studi di Milano. Sample CE-1 was collected in core Ceprano 1 at 39.3 m depth within a coarse gravel layer with abundant sand matrix. The youngest crystal out of a population of 30 extracted from this sediment yielded a ^40^Ar/^39^Ar age of 452.4 ± 1.8 ka. Sample CE-2 was collected at 15.1 m depth in borehole Ceprano 2, at the base of a coarse sand layer and yielded a youngest crystal date of 389.6 ± 3.7 ka. As discussed in the previous section, these ages can be considered good approximations of the time of deposition of these sediments (see also “Age interpretations”).

**Figure 4:**
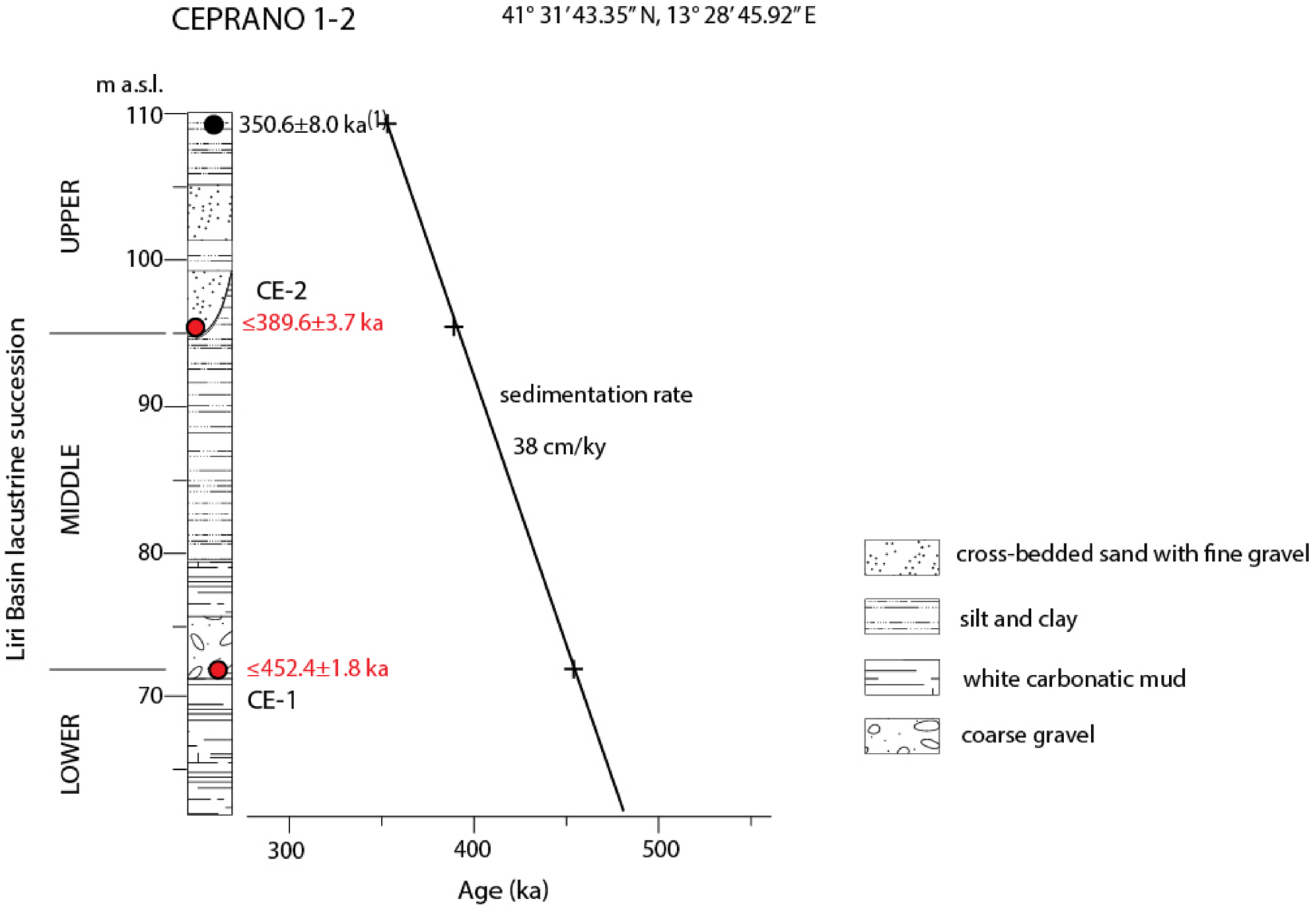
Synthetic stratigraphic log of the two boreholes drilled in Ceprano^22^ showing the samples dated in the present work (red dots) and in Nomade et al.^33^ (black dot), which allow to assess the sedimentation rate of the lacustrine succession.

Consistent with this hypothesis, a constant sedimentation rate of ~ 38 cm/ky is calculated from our sample ages combined with that of 350.6 ± 8.0 ka^33^ on the primary volcanic layer cored at the top of the lacustrine succession (Fig. 4), in good agreement with previous estimation (30–40 cm/ky^22^). While linear long-term sedimentation rates are likely to obscure high frequency changes in sediment accumulation as revealed by sedimentological observations, they provide useful constraints on the accumulation history of the basin during the overall lifetime of its existence. Indeed, gravel and cross-bedded sands represent sudden, high-energy inputs within the lacustrine basin. Also in consideration of their small thickness with respect to the silty-clayey sediments, the ages of the samples collected in these two horizons may be regarded as good spot constraints to the overall lacustrine sedimentation, which is characterized by relatively constant sedimentation rate.

These observations indicate that the ages of 452.4 ± 1.8 ka and 389.6 ± 3.7 ka are excellent time constraints on the deposition of the gravel horizon at the base of the "Middle lacustrine-fluvial succession", and to the start of coarse-sized sedimentary input (cross-bedded sands with sparse gravel) at the base of the "upper fluvial-lacustrine succession".

#### Cava Pompi

Temporary archaeological trench excavations at this location^34^ exposed a ca. 3 m thick sedimentary succession overlying a primary pyroclastic-flow deposit (Suppl. Fig. S1). It was constituted, from bottom to top, by a basal, coarse sand-and-gravel volcaniclastic horizon, mostly deriving from reworking of the underlying volcanic deposit. It abruptly passed upwards to bedded, white lacustrine muds and massive, yellowish sandy silt with clay intercalation. A travertine horizon closes the succession.

Lower and upper age constraints to this sedimentary succession are provided by two ^40^Ar/^39^Ar ages performed on the pedogenically weathered top horizon of the pyroclastic flow deposit occurring at the base of the succession^35^, and on a second, primary volcanic deposit (this work) unconformably overlying it (Suppl. Fig. S1). The lowest sample provides a *terminus post-quem* of 394.3 ± 8.2 ka to the beginning of sedimentation, while the upper sample (PO-C6) gives a *terminus ante-quem* of 337.5 ± 6.4 ka for it.

#### Colle Avarone

A ca. 8 m-thick succession is exposed at several sections at this locality (Suppl. Fig. S2). A coarse gravel in abundant sand-matrix horizon, > 2 m in thickness, occurs at the base and is overlain by a lacustrine succession in which three primary volcanic deposits are intercalated. Two ^40^Ar/^39^Ar ages on one sample (CA-CGT) of sand matrix collected in the basal gravel horizon and on the uppermost pyroclastic-flow deposit (CA-C1) were performed^21^, bracketing the deposition of the sedimentary succession between 359.6 ± 6.5 and 345.9 ± 4.3 ka.

#### Isoletta

A more than 30 m-thick section was temporarily exposed during construction of the high-velocity railway in the 90's at this location^36^. A more than 10 m-thick grey clay-and-silt lacustrine to fluvial deposit was exposed at the base of the succession, followed by another ca. 10 m-thick package of cross-bedded to planar coarse sand (Suppl. Fig. S3). A ca. 3 m-thick layer of coarse gravel follows in the succession, transitioning upwards to sand and silt. A travertinaceous silt horizon closes the succession. Two sub-primary (i.e., reworked, in-place ashfall deposit) volcanic layers intercalated at the base of the clay and in the middle of the coarse sand horizons provided ages of 400.3 ± 3.0 and 372.2 ± 2.8 ka, respectively^35^. A reworked volcaniclastic layer collected in the coarse gravel horizon yielded a youngest population of two crystals providing a *terminus post-quem* age of 362.6 ± 3.8 ka for its deposition^35^.

#### Lademagne

The sedimentary succession is made up of a 2 m-thick horizon of sand with abundant medium-to coarse gravel, overlying a clay layer and topped by 1 m of sandy silt deposits and was described at this location^37^. Two sub-primary volcaniclastic layers collected in the vicinity of the original archaeological site, which stratigraphically constrain the sedimentary succession at the bottom and at the top (Suppl. Fig. S4), yielded ages of 402.4 ± 4.8 ka and 386.2 ± 4.6 ka, respectively^35^.

#### Pontecorvo

Two tephra layers interbedded in the carbonate-rich muds of the "Lower Lacustrine Succession" of the Liri basin cropping out in the surroundings of Pontecorvo village were dated by the K/Ar method at 583 ± 11 ka and 570 ± 11 ka^19,38^. We have re-investigated this sector and detected a geologic section in which several tephra layers occur at elevation ranging 55–57 m a.s.l. (Suppl. Fig. S5).

#### San Giorgio al Liri

**Figure 5:**
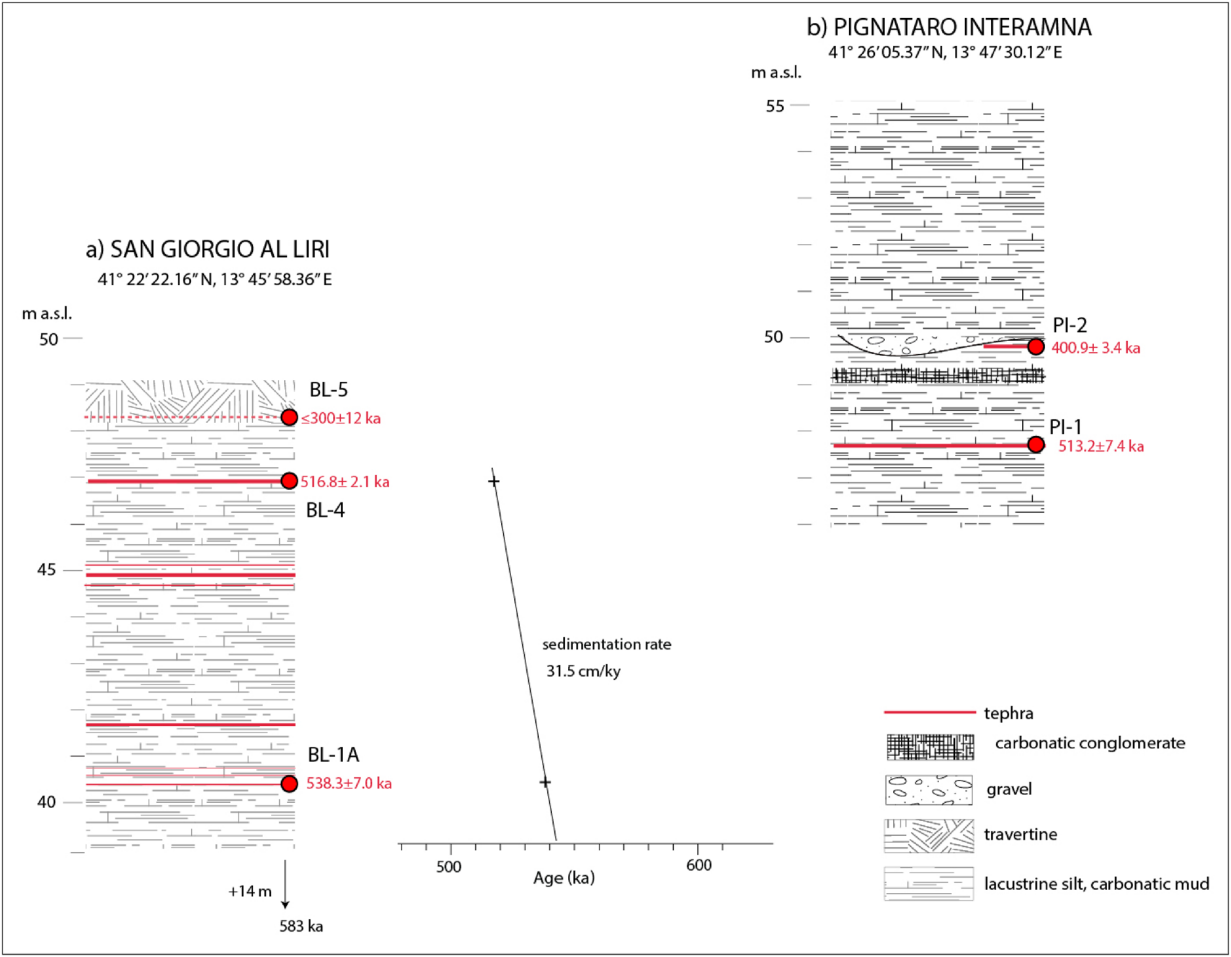
Stratigraphic sketches of San Giorgio al Liri (**a**) and Pignataro Interamna (**b**) sections showing the sampled tephra layers and the average sedimentation rate.

A more than 10 m-thick succession of white carbonate-rich muds, silts and travertine layers cropping out in the neighborhoods of San Giorgio al Liri village was discovered (Fig. 5a). Eight cm- to dm-thick tephra layers are intercalated in the lowest 8 m of the succession which is constituted by lacustrine white carbonate-rich muds. We have dated the lowermost (BL-1A) and uppermost (BL-4) of these tephra layers, which yielded ^40^Ar/^39^Ar ages of 538.3 ± 7.0 ka and 516.8 ± 2.1 ka, respectively, allowing correlation of the sedimentary deposits with the "Lower lacustrine succession". In contrast, a sedimentary sample collected in the travertine horizon at the top of the succession provided a *terminus post-quem* age of 300 ± 12 ka, evidencing a large sedimentary hiatus at the top of the lacustrine succession.

The ages of the two tephra layers allow an estimate of the sedimentation rate of ca. 31.5 cm/ky for the lacustrine basin during the corresponding time span.

#### Pignataro Interamna

A more than 20 m-thick lacustrine succession crops out in the surroundings of Pignataro Interamna village, which was attributed to the "Typical lacustrine facies"^19^. We have investigated the lowest, 10 m-thick portion of this succession cropping out few km southeast of Pignataro Interamna, constituted by white carbonate-rich muds and yellow silts, in which two clastic horizons are intercalated (Fig. 5b). We sampled and dated one primary tephra occurring in the lower part of the lacustrine deposits and the volcaniclastic sand matrix of a discontinuous, up to 20 cm thick gravel layer occurring in the upper portion of the succession. An anomalous, 50 cm-thick conglomeratic horizon constituted by poorly rounded, ≤ 1 cm sized carbonate fragments, chert and limestone pebbles within a silty matrix occurs ca. 1.5 m above the lowest tephra layer dated at 513.2 ± 7.4 ka (sample PI-1). The maximum age of 400.9 ± 3.4 ka provided by the sample PI-2 collected in the upper gravel layer, 50 cm above the conglomerate, implies that this clastic horizon marks a significant sedimentary hiatus, similar to that occurring at the top of the "Lower lacustrine succession" in San Giorgio al Liri. However, more lacustrine deposits occur above this hiatus in Pignataro Interamna, which are a lateral transitional facies of the "upper fluvial-lacustrine succession", as evidenced by the geochronologic constraints provided here.

## Discussion

### Age interpretations

Hereby we discuss the interpretation of the maximum ages derived from dating of four sedimentary samples: CA-CGT (dated in Marra et al.^21^), CE-1, CE-2, BL-5 (dated in the present study).

#### Sample CA-CGT

**Figure 6:**
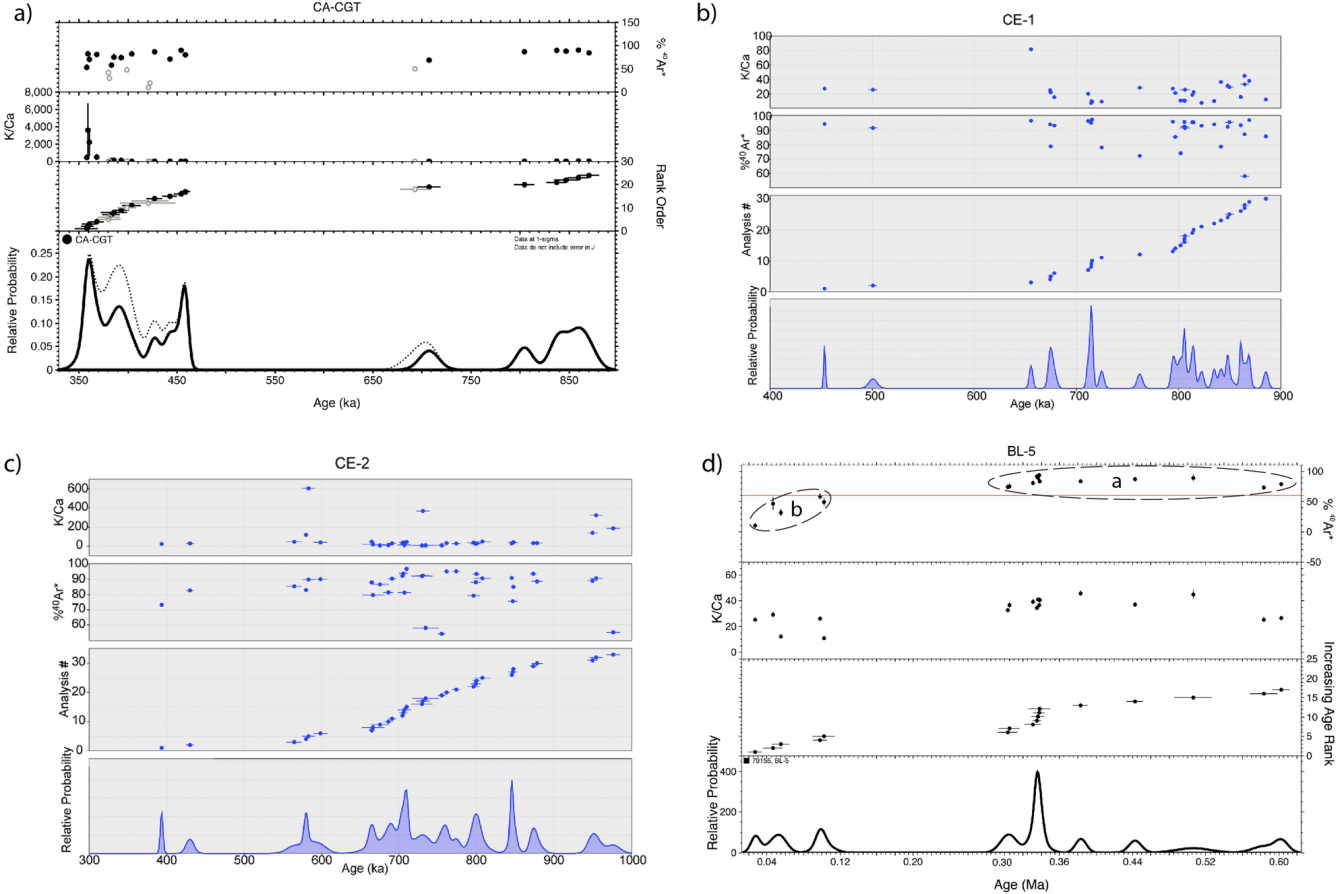
Results of ^40^Ar/^39^Ar analyses on single crystals presented as probability diagrams along with plots of %^40^Ar* and K/Ca ratios. (**d**) Age populations of sample BL-5 are further distinguished by the amount of radiogenic Ar (%40Ar*); see text for comments and explanation.

24 crystals extracted from the sand matrix of a ca. 2 m thick gravel layer cropping out at Colle Avorone locality (Sample CA-CGT) were dated by Marra et al.^21^. 3 youngest crystals provided a weighted mean age of 359.5 ± 6.5 ka (2σ uncertainty) (Fig. 6a). Other 21 crystals provided two groups of ages, the largest one (14 grains) ranging 359–450 ka, with other six crystals spanning 700–850 ka (Fig. 6a).

The younger group of ages matches the duration of the main eruptive phase occurred at the Volsci Volcanic Field (VVF) 424 ± 13–349.5 ± 5.0 ka^21^.

Regarding the older crystal ages, it should be noted that five samples from phreatomagmatic deposits analyzed in Marra et al.^21^ yielding weighted mean ages in the interval 761.5 ± 9.5 to 349.5 ± 5.0 ka evidenced significant dispersion towards old ages. These products were mainly sourced from isolated, monogenetic eruptive centers. Therefore, it is unlikely that the older age values might have derived from earlier buried volcanic edifices or erupted deposits, and yet may provide evidence of older magma batches that cooled in sub-surface conditions.

#### Samples CE-1, CE-2

Sample CE-1 was collected in borehole core Ceprano 1 at 39.3 m depth within a coarse gravel layer with abundant sand matrix. The youngest crystal out of a population of 30 extracted from this sediment yielded a ^40^Ar/^39^Ar age of 452.4 ± 1.8 ka (Fig. 6b). Sample CE-2 was collected at 15.1 m depth in borehole Ceprano 2, at the base of a coarse sand layer and yielded a youngest crystal date of 389.6 ± 3.7 ka (Fig. 6c).

These two maximum ages can be regarded as statistically significant even if based on one single crystal. The Ceprano boreholes were drilled in Campogrande which is located on the left hydrographic side of the Sacco catchment basin, a sector draining the most active and densely vent-populated volcanic area of the Volsci Volcanic Field. A climactic eruptive phase occurred at the VVF in the interval 420–350 ka^21^, so the lack of crystals younger than 453 ka strongly suggests that the emplacement of the sand deposit occurred before the start of this volcanic phase. Consistent with this hypothesis, there is one crystal of 428 ± 10 ka along with one youngest crystal of 390 ± 3.6 ka in the sample stratigraphically above. Moreover, these two ages along with that of 350.8 ± 8 ka on the primary layer occurring at the top of the sedimentary succession recovered in the Ceprano boreholes^33^ provide a constant sedimentation rate of 38 cm/ky (see Fig. 4).

Therefore, it is reasonable to assume that likewise, the maximum ages derived from reworked sanidine crystals can be regarded as providing precise time constraints to sediment deposition, as the one on the primary volcanic layer.

Indeed, an age close to 453 ka for the gravel deposition during MIS 11 is in good agreement with the constraints provided from the Paleo-Tiber aggradational successions, which bracket it between 451 ± 2 and 445 ± 3 ka^10,13^.

#### Sample BL-5

Sample BL-5 was collected in a sandy-clayey travertine layer embedding several sub-cm sized, very altered volcanic scoriae. Crystals extracted from this sample yielded two distinct groups of ages ("a" and "b" in Fig. 6d).

One group of older ages ranging 300–600 ka ("a") is consistent with expected distribution for a deposit reworking the volcanic deposit of the Volsci Volcanic Field (VVF), the activity of which broadly spanned the interval 750–250 ka, as also observed in the sedimentary samples CE-1 and CE-2 (see Fig. 6b,c). The occurrence of one second group of ages ranging 120–30 ka ("b") is problematic to explain, given the much unlikely circumstance that it may reflect the real age of the deposit. Indeed, the travertine layer occurs on top of the "lower lacustrine succession", closely constrained at this location by tephra ages of 538 and 517 ka, and is part of the "upper fluvial-lacustrine succession" which previous^19^ and the present study constrain between 390 and ca. 300 ka.

Therefore, two hypotheses can be made to explain the second group of crystal ages. The anomalously young ages most probably represent much younger ash fall and/or eolian material that was worked into the deposit through fractures; indeed, the sampled deposit was exposed on a slightly inclined surface in the middle of a hillside, which is affected by continuous sliding of reworked sediments from the uphill section. Alternatively, these anomalous young ages may result from contamination in the field or laboratory. In any case, we consider the weighted mean age of 300 ± 12 ka yielded by the two youngest crystals in group "a" as a reliable maximum age for the deposit.

### Gravel origin and mechanisms of transport and deposition

The Liri basin is a lacustrine basin of tectonic origin^24,25^, similar to other intra-mountain basins in the central Apennines^39^, like the Fucino^40^, L'Aquila^41^, Sulmona^42^, and Anagni^43^ basins, characterized by deposition of thick successions of carbonate muds^43^. The occurrence of discrete gravel beds in the Sacco-Liri basin were interpreted in the literature (e.g., Devoto^19^) as the evidence of intervening fluvial conditions. Our study shows that conglomerates represent episodes of high-energy water transport superposing the lacustrine conditions. The correlation of the investigated cross-sections shows that there are only two large gravel beds in the basin, which can be correlated laterally for several ten kilometers, intercalated with lacustrine muds. On the contrary, local mechanisms for gravel deposition, like occasional damming, may cause deposition of very limited fans. Moreover, in order to have gravel deposition as a consequence of damming, there must be strong capacity of transport within the catchment basin, which is not the case for a lacustrine basin such as the Sacco-Liri basin.

In contrast, during deglacial periods there is a strong release of gravel from the fans at the foot of the carbonate reliefs that border the upper portion of the catchment basin. This gravel is mobilized by the strongly increased water capacity of transport due to the increase in rainfall during the glacial termination, at the end of the cold-arid period leading into the warm-humid period.

Such processes have been documented and dated through the aggradational successions of the Tiber River catchment basin, which responded to variation in sea level. In contrast, an intra-mountain lacustrine basin is not expected to respond to the variation in base level, directly. However, during sea-level low stands an increased gradient in the lower portion of the basin (between the sea and the Liri basin) may cause erosion of the lacustrine deposits, with a shift into fluvial conditions which reverberates in the higher portion of the catchment basin, also causing an increase in the gradient. This adds increase in water capacity of transport allowing gravel eroded from the mountain slopes to be deposited in the Liri basin during the glacial termination at the beginning of the sea-level rise, when the baselevel is still low.

^40^Ar/^39^Ar ages provided here to constrain the gravel deposits demonstrate that their emplacement is coeval with global deglacial episodes, as reported in the δ^18^O curve^44^.

### Morphostructural analysis of the Liri basin

**Figure 7:**
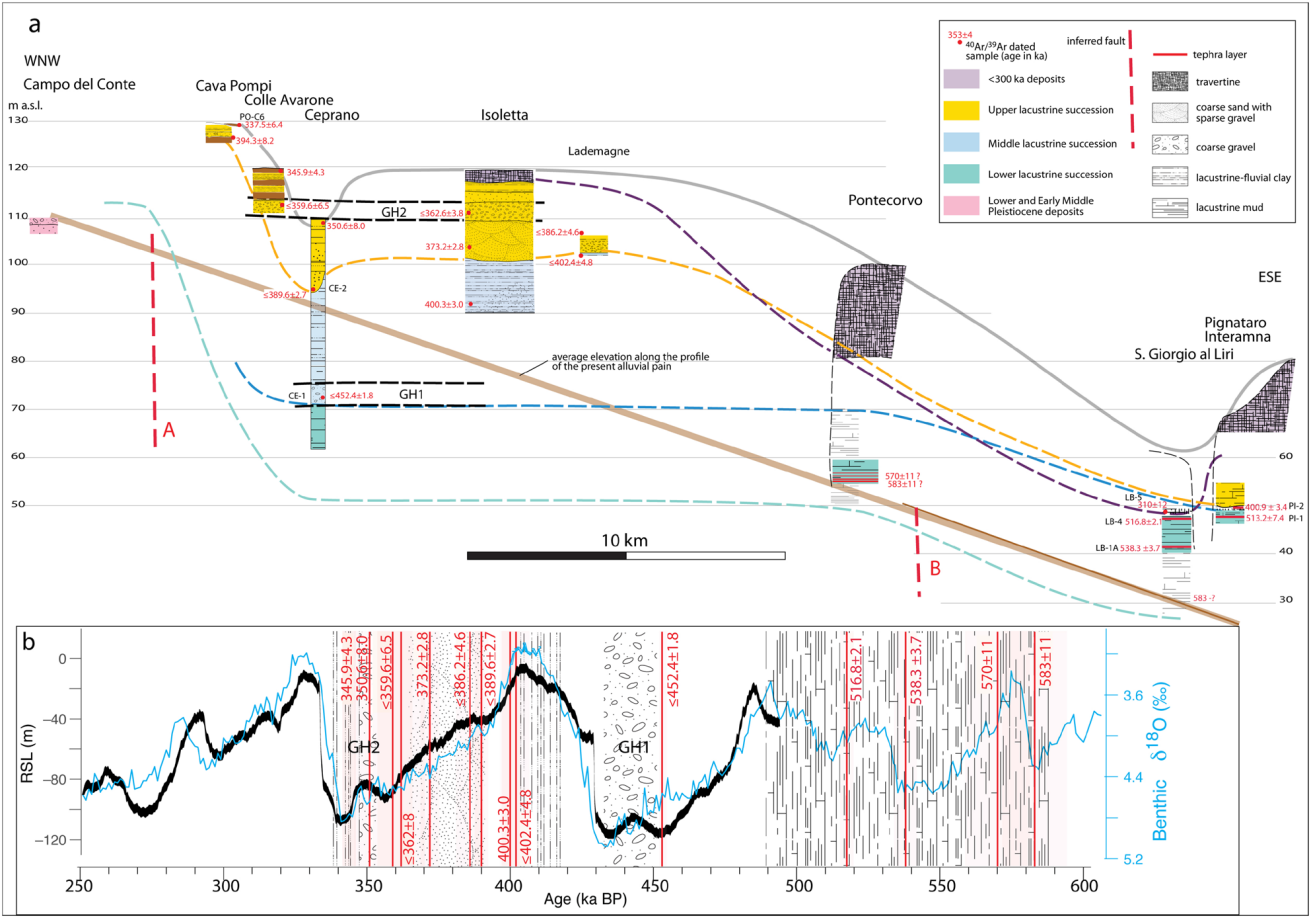
(**a**) Schematic cross-section along the Liri basin showing the chronostratigraphic correlation of the investigated sections. See text for comments and explanation. (**b**) The age constraints to the sedimentary filling of the Liri basin provided by volcanic layers dated in this and in previous works (vertical red bars; shaded boxes are the 2σ uncertainties) allow comparison of sediment aggradation with the stack of δ^18^O records^44^ and the relative sea level (RSL) curve^45^.

**Figure 8:**
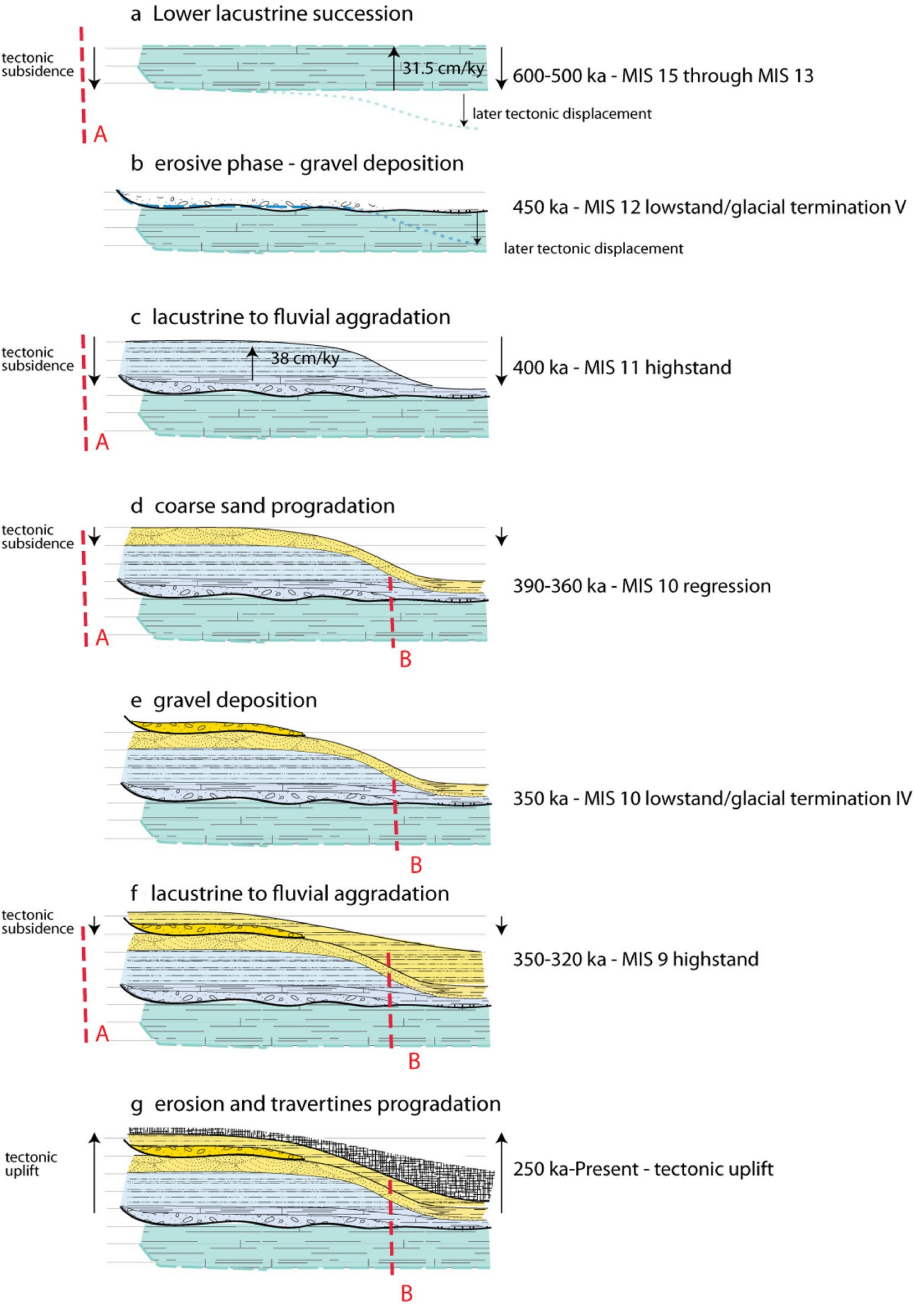
Proposed stepwise reconstruction of the sedimentary and tectonic evolution of the Liri basin through time. See text for comments and explanations.

Figure 7A shows a NNW-SSE cross-section along the Sacco-Liri valley providing the chrono-stratigraphic correlation of the investigated sections, while a tentative reconstruction of the structural and sedimentary evolution of the Liri lacustrine basin is provided in Fig. 8.

Correlation of the sedimentary phases with the stack of globally distributed δ^18^O records^44^ and the Red Sea relative sea-level (RSL) curve^45^ is shown in Fig. 7b.

The occurrence of a Lower Pleistocene succession in Campo del Conte at higher elevation with respect to the Middle Pleistocene lacustrine succession in the Liri Basin provides a paleogeographic constraint to the tectonically subsiding sector (inferred fault A in Figs. 2 and 8a). Consistent with the present morphology of the Sacco Valley, confined within a narrow incision between the Meso-Cenozoic sedimentary and Middle Pleistocene volcanic terrains (dark green bordered sector in Fig. 2), no significant lacustrine sediments occur in this area, showing the absence of tectonic subsidence. In contrast, a more than 50 m-thick sedimentary succession spanning 450–337 ka (middle and upper lacustrine and fluvial successions) occurs in the sector between Cava Pompi and Lademagne, broadly corresponding to the Liri lacustrine basin (light green bordered sector in Fig. 2), and overlies more lacustrine sediments (lower lacustrine succession), with ages spanning at least the 583–515 ka interval (Fig. 7a).

Extensive stratigraphic investigations conducted for the present work, summarized in the cross-section of Fig. 7, showed that the presence of coarse gravel beds cannot be related to local factors but concentrate in two distinct stratigraphic horizons. The uppermost of this horizon has average thickness of ca. 3 m over a large areal extent, covering at least 10 km, between Campo Grande and Lademagne (see Fig. 2), where it crops out at constant elevation between 110 and 115 m a.s.l. The lower gravel bed occurs at lower elevation, between 70 and 75 m a.s.l. in the Ceprano boreholes and is intercalated within the clayey sediments of the "Typical lacustrine succession" as far as Pontecorvo^19^.

Taking into account all the above-mentioned chrono-stratigraphic constraints, we reconstructed a simplified structural evolution for the Liri Basin (summarized in Fig. 8).

A lower age constraint to the initiation of the tectonic subsidence of the Liri Basin is not available because the total thickness of the lower lacustrine succession is not known.

In Fig. 8a,b, we have represented only the portion of the lower lacustrine succession which has chronostratigraphic constraints, between 583 and 515 ka. Given the peculiar sedimentologic features of the lacustrine sediments, represented by carbonate-rich muds which are characteristic of a shallow water environment^19^, this succession should have deposited at relatively constant elevation throughout the Liri basin (Fig. 8a). There is no reason to exclude that deposition continued until 500 ka (MIS 13.3 highstand, Fig. 7b) and that the upper portion of the succession was successively eroded during the MIS 12 regressive phase (Fig. 8b), as evidenced by the stratigraphy of the Pignataro Interamna section and by the age constraint of 452.4 ± 1.8 ka (sample CE-1) from the lower gravel horizon in the Ceprano boreholes (GH1 in Fig. 7a).

A close relation between the occurrence of MIS 12 lowstand and the deposition of the gravel layer GH1 at the base of the middle lacustrine-fluvial succession is evidenced by the age of sample CE-1 (Fig. 7b). ^40^Ar/^39^Ar constraints to the upper portion of this succession show that rapid aggradation of lacustrine to fluvial clay sediments occurred since the Glacial Termination V, which has an established age of 424 ka^44^, through 402 ka in response to MIS 11 sea-level rise. The increased sedimentation rate based on the ^40^Ar/^39^Ar age constraints at the Ceprano boreholes is suggestive of enhanced tectonic subsidence in coincidence with the climactic eruptive phase occurred at the Volsci Volcanic Field from 424 through 350 ka^21^. Starting from 390 ka, an increase in water transport energy within this sector of the Liri catchment basin resulted in an abrupt sedimentary switch, leading to the deposition of coarse sand with intercalated fine gravel sediments (Fig. 8d; upper fluvial-lacustrine succession). It is followed by the deposition of a 2–3 m thick horizon of well-sorted, coarse gravel, narrowly constrained in the interval 350.6 ± 8.0–345.9 ± 4.3 ka. This time interval encompasses the entire regressive phase of MIS 10 (Fig. 8e). Another abrupt sedimentary shift into clayey sediments is noted at Colle Avarone and Cava Pompi sections, where lacustrine deposits overly the coarse gravel horizons (Fig. 8f). However, thickness of the fine-grained portion of the upper fluvial-lacustrine succession is very limited and it passes upwards to wide travertine plateaus characterized by very thick deposits to the southeast^19^ (Fig. 8g).

We correlate this sedimentary change to the decrease/cessation of the tectonic subsidence in the Liri Basin, which may be due to the vanishing of the volcanic activity at the Volsci Volcanic Field (VVF) since 350 ka. Published ages of the VVF spanning 300–200 ka are poorly constrained, with the youngest reliable eruption age that of the Colle Borrello center occurring at 331.6 ± 3.0 ka^21^. The lack of a fine-grained aggradational succession deposited during MIS 9 highstand (337–325 ka, Fig. 7b) is likely related to the tectonic inversion within the Liri Basin.

Evidence for deep erosion of the upper fluvial-lacustrine succession occurs at San Giorgio al Liri section, where travertine deposits directly overlying the lower lacustrine succession yielded a *terminus post-quem* age of 300 ± 12 ka, consistent with occurrence of the regressive phase of MIS 8 since 320 ka (Fig. 7b). Contextual erosion and travertine progradational deposition is likely to have occurred throughout MIS 8, 320–270 ka (Fig. 7b), while the origin of a series of terraced paleo-surfaces throughout the Liri Basin is reflecting the subsequent regional uplift phase. Indeed, according to several authors^46–50^, the Tyrrhenian Sea Margin of central Italy underwent variable uplift of several tens of meters in the last 250 ka.

### Relationships between sediment grainsize and sea-level fluctuations

The ^40^Ar/^39^Ar constraints provided here to the deposits of the middle lacustrine succession bracket their deposition in the interval 452.4 ± 1.8 to 400.3 ± 3.0 ka, demonstrating that they correspond to an aggradational succession sensu Marra et al.^5,7^. In other words, the dated morpho-sedimentary unit formed in response to sea-level rise during the glacial–interglacial transition.

The complete chronostratigraphic record of the MIS 11 aggradational succession occurring in the coastal setting (San Paolo Formation^2,7,10,13,14^) reported in Fig. 8a shows that the equivalent deposit of the Liri Basin provides indistinguishable age constraints to the deposition of the basal gravel layer and to the completion of aggradation of the fine-grained sedimentary package, between 450 and 400 ka.

**Figure 9:**
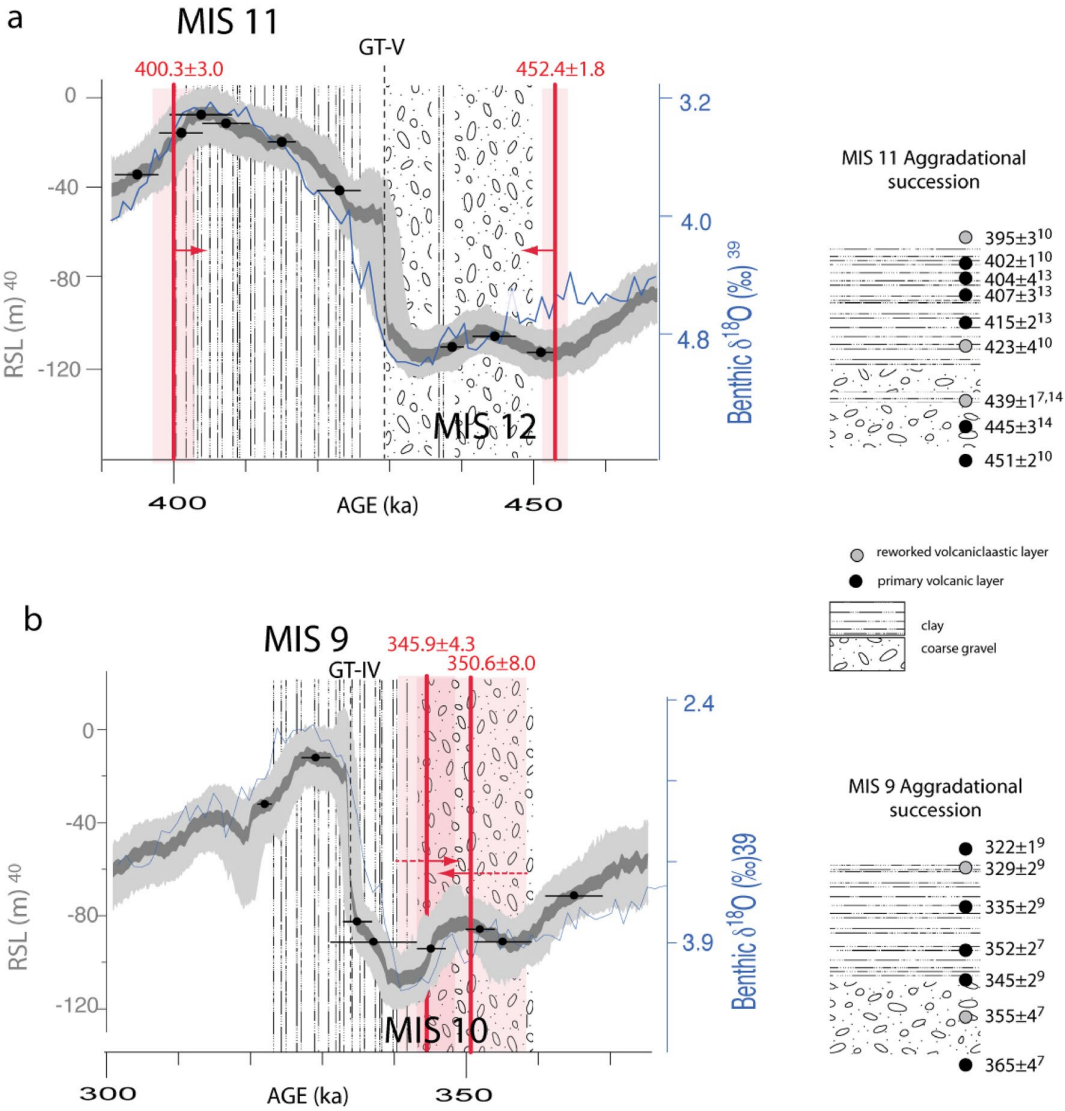
^40^Ar/^39^Ar age constraint to sediment aggradation in the Liri basin (red vertical bars) for (**a**) MISs 11–12 across Glacial Termination V, and (**b**) MISs 9–10 across Glacial Termination IV are compared to those provided in previous works to the aggradational successions of the Paleo-Tiber River reported in the stratigraphic sections to the right. Red arrows pointing to the left indicate terminus post-quem (maximum) ages and those pointing to the right indicate terminus ante-quem (minimum) ages. See text for comments and explanation.

Remarkably, Giaccio et al.^14^ demonstrated the early aggradation of a first gravel layer at 443.1 ± 3.2 ka before the onset of Glacial Termination V and coinciding with a minor sea-level rise on the RSL curve^45^ (Fig. 9a). Following this event, another gravel layer passing upwards to a thick clay succession was deposited after 437.1 ± 1.2 ka, in good agreement with the timing of the glacial termination and the sea-level rise at the onset of MIS 11 highstand (Fig. 9a). Giaccio et al.^14^ interpreted the early aggradation phase during MIS 12 as a first meltwater pulse (MWP1) event, preceding the larger amplitude meltwater pulse during Glacial Termination V (MPW2). These authors remarked that both MWPs coincide with episodes of ice-rafted debris deposition in the North Atlantic (Heinrich-like events^51^) and with attendant Southern Hemisphere warming, plausibly associated with the bipolar seesaw. Indeed, the occurrence of prominent ice-rafted debris (IRD^18^) peaks associated with these meltwater pulses alludes to episodes of extensive iceberg calving in the North Atlantic, consistent with sustained melting of the circum-North Atlantic ice sheets^52^ during these events.

The age of 452.4 ± 1.8 ka, which constrains the start of gravel deposition in the Liri Basin, provides strong indication of coarse clastic input to the river catchments of the Tyrrhenian Sea margin deriving from early deglaciation in the Apennine mountain range. It also provides further evidence for the validity of the sedimentary model of the "aggradational successions"^5,7^.

The reasons for such a far-field response also rely on the lesser elevation gain within the catchment basin, which has been successively increased by regional uplift over the last 250 ka^46–50^. In contrast, a comparison of the average uplift rate in the last 250 ka (0.24 mm/year) with the average sedimentation rate during the aggradational phases (e.g., 2.3 mm/year) suggests that glacio-eustasy overrides the tectonic effects, which only impact the accommodation space, and, in turn, the total thickness (rather than the timing of deposition) of each aggradational succession^9^.

The early aggradation of the MIS 9 sedimentary record (Aurelia Formation^2^) has been presented^7^ and has been constrained further by successive work^9^. In particular, a primary volcanic deposit dated at 345 ± 3 ka at the top of the basal gravel bed of the MIS 9 aggradational succession in the upper sector of the Tiber River catchment basin^9^ (Fig. 9b) provided a *terminus ante-quem* for its deposition, preceding the canonical age of 337 ka^44^ for Glacial Termination IV. The narrow age constraints to the second gravel layer occurring in the upper lacustrine succession in the Liri Basin provide a striking match with those provided in the coastal and more inner sectors of the Tiber River basin^2,7,9^, evidencing an early deglaciation occurring during MIS 10 (Fig. 9b). Remarkably, this early aggradational phase also corresponds with a minor sea-level rise in the RSL curve preceding the glacial termination (Fig. 9b), suggesting the same triggering mechanism (i.e., early ice melting) as that hypothesized for the analogous eustatic event during MIS 12.

### Possible triggering mechanisms to deglaciation

The occurrence of meltwater pulses/events during the glacial maxima that preceded both T-V and T-IV is challenging since the two glacial–interglacial cycles have very different glacial histories and insolation forcing (e.g., Spratt and Lisiecki^53^). Moreover, T-V was characterized by slower rates of ice-sheet melting/sea-level rise than for most of the last five terminations^45^.

While an in-depth analysis of the possible causes is beyond the scope of this paper, we remark on an intriguing aspect of the insolation curve during these glacial termination which was already pointed out as a possible contributing factor to trigger the deglacial process.

**Figure 10:**
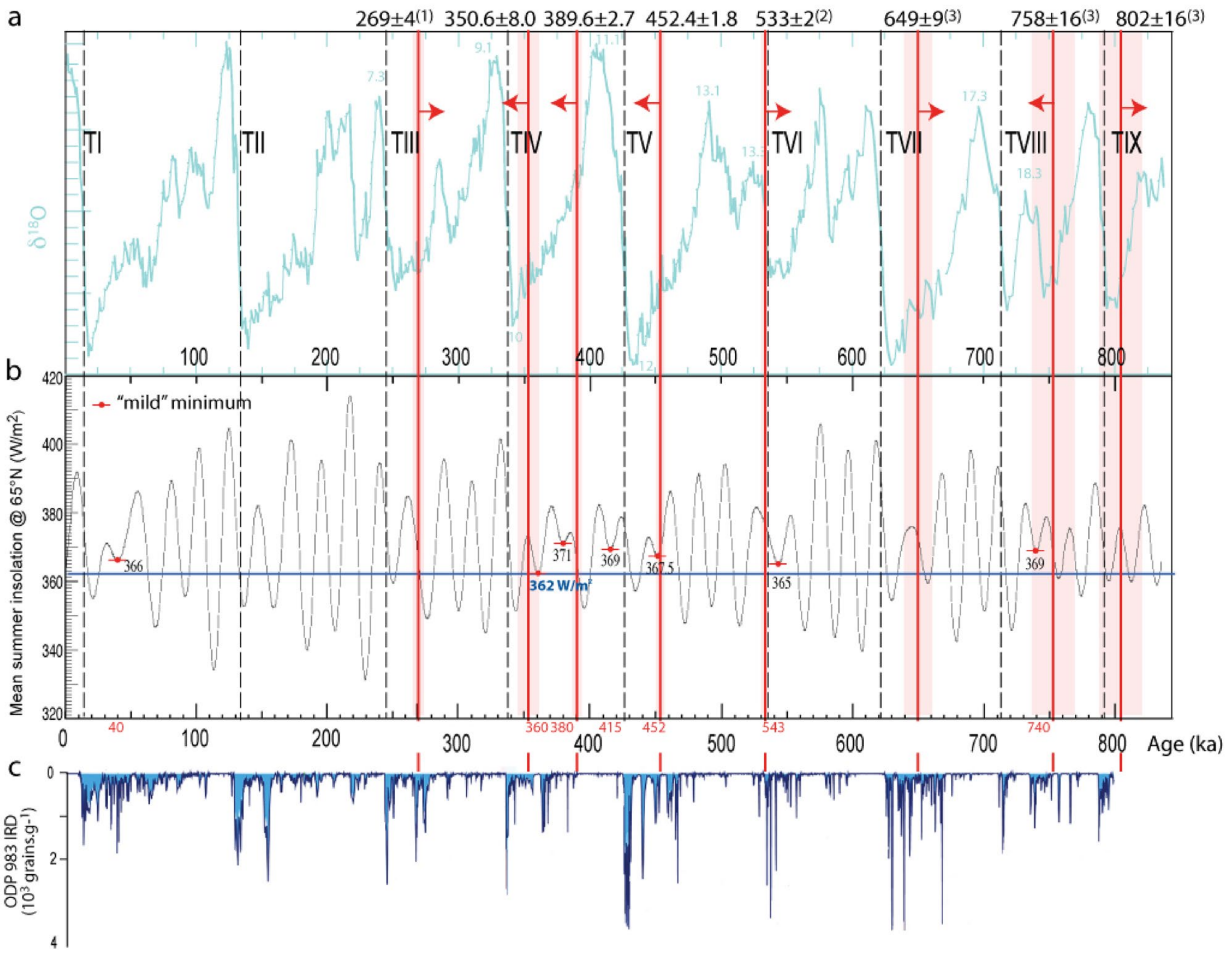
^40^Ar/^39^Ar age constraints (vertical red bars, shaded boxes are the 2σ uncertainties) to the coarse gravel beds occurring in the sedimentary filling of the Liri basin provided in this work, as well as at the base of the aggradational successions of the Paleo-Tiber River provided in previous studies^7,8,11^. Red arrows as in Fig. 8. (**a**) Comparison with the glacial termination (GT) ages established through the calibration of the δ^18^O curve^44^; (**b**) comparison with a set of "mild" minima (see text for explanation) of the mean summer insolation curve at 65° N^54^; (**c**) comparison with the ice-rafted debris (IRD) record of core ODP 983^51^ (re-drawn here for comparative purpose).

Associated with the introduction of the aggradational successions model, Marra et al.^7^ proposed a possible forcing mechanism based on the occurrence of particularly mild insolation minima which may be regarded as the pre-conditioning factor to trigger a glacial termination. We re-propose this notion (Fig. 10) based on the comparison of the two geochronologically constrained sedimentary records of glacial terminations V and IV provided in this paper with the mean summer insolation curve at 65° N^54^.

The interval 450–359 ka encompassing the two glacial terminations investigated in this paper is characterized by the occurrence of four such "mild" minima. Indeed, if we consider the insolation value of 362 W/m^2^ for the insolation minimum occurring at 360 ka as a lower threshold (blue horizontal line in Fig. 10), we see that only six other minima were characterized by higher insolation, whereas all other minima in the last 800 ky were "colder". These six mild (warmer) minima are all associated with evidence of anomalous meltwater pulse events. The oldest one (369 W/m^2^), at ca. 740 ka, is associated with the early emplacement at ca. 750 ka of a thick gravel layer in the Paleo-Tiber delta, which was interpreted as a previously unrecognized glacial termination (i.e., MIS 18.3, TVIII-A^4,5^). Remarkably, the second oldest mild minimum at 543 ka is associated with an identical feature of the δ^18^O curve: a double isotopic peak. However, the only difference in this case is that the earliest peak (13.3) is associated in the literature with the eustatic event considered to be the true glacial termination VI, as opposed to MIS 17, for which the glacial termination has been associated with the younger 17.3 peak^44^ (Fig. 10). Unfortunately, the lack of a detailed record of RSL in the interval > 500 ka hinders the possibility to verify if both these mild minima actually preceded meltwater pulses.

In contrast, a striking coincidence between the mild minima at 452 and 360 ka and the early aggradational phases during MIS 12 and MIS 10, which are associated with the emplacement of the coarse gravel beds at ~ 453 and ~ 351 ka in the catchment basins of the Tiber and of the Sacco-Liri rivers, is evident by inspection of Fig. 10. A lesser of such events is also clearly associated with the mild minimum of 371 W/m^2^ occurring at 380 ka, which triggered the grainsize inversion in the Liri Basin causing the emplacement of the coarse sand-and fine gravel horizon which has a *terminus post-quem* age of 390 ± 1 ka.

Moreover, the most outstanding observation is that the only other mild minima of 366 W/m^2^ in the last 350 ka occurred at 40 ka, in close connection with the onset of the Heinrich events^51^.

This is strong supporting evidence for relating the early aggradational phases responsible for the emplacement of gravel beds in the Tiber and Liri basins with deglaciation events (meltwater pulses) that triggered relatively minor sea-level rises, and which represent the equivalent of past Heinrich-like events in the Apennines of central Italy.

## Conclusions

^40^Ar/^39^Ar geochronology used to constrain the aggradational phases and grainsize variations of the sedimentary filling of the Liri fluvial-lacustrine basin provide strong evidence to support the "aggradational successions" model^5,7,14^ as a powerful tool to detect the occurrence of deglaciation events that triggered global meltwater pulses.

We demonstrate a substantial synchronicity between the ages of gravel deposition in both the Tiber and Liri rivers catchment basins and the occurrence of moderate sea-level rise events, which anticipate those more marked during the glacial terminations V and IV in the Red Sea relative sea level curve^45^.

We also show a striking correspondence among the occurrence of particularly mild (warmer) minima of the mean summer insolation at 65° N^54^ and these early aggradational phases, as well as with other anomalous early sea-level rises occurring at the onset of glacial terminations VIII and VI, and at 40 ka at the onset of the so-called Heinrich events^51^.

Such patterns suggest that gravel deposition is triggered by melting of the Apennines mountain range glaciers, providing the water transport energy and a surplus of clastic input in the catchment basins of the rivers draining the mountain regions and flowing into the Tyrrhenian Sea. Such hydrologic/sedimentary processes, which occur in any mountain region of the globe, if correctly depicted and dated, can provide a large dataset of deglaciation proxies to unravel the chronology of glacio-eustatic events occurring in the last 1 Ma.

## Methods

### ^40^Ar/^39^Ar analysis

Samples for ^40^Ar/^39^Ar analyses were prepared at the Laboratoire des Sciences du Climat et de l’Environnement facility (CNRS-CEA, Gif-sur-Yvette), France, and at the University of Wisconsin-Madison.

Three distinct irradiations have been performed and the samples were dated in three facilities (Berkeley Geochronology Center, USA), Laboratoire des Sciences du Climat et de l’Environnement (CEA, Gif-sur-Yvette), and WiscAr Laboratory of Wisconsin University (USA). Samples PO-C6, BL-1A, BL-5, PI-1 and PI-2 were irradiated in the Cd-lined, in-core CLICIT facility of the Oregon State University TRIGA reactor.

Samples PO-C6, BL-1A and BL-5 were analyzed at the Berkeley Geochronology Center (BGC; California, USA), using a MAP 215-C mass spectrometer (MAP 1), following procedures described in Giaccio et al.^55^. Samples BL-4, CE-1, and CE-2 were analyzed at the University of Wisconsin-Madison (USA), using a Noblesse 5-collector mass spectrometer, following procedures described in Jicha et al.^56^. Samples PI-1 and PI-2 were analyzed at LSCE using a VG 5400 mass spectrometer (LSCE; Gif-sur-Yvette, France), following procedures described in Pereira et al.^35^.

All ages are calculated according to the fluence monitor age of Alder Creek sanidine (40 K total decay constant of Min et al.^57^; ACs = 1.1848 ± 0.0012 Ma^29,30^) and are reported to the precision level of 2σ standard deviation. Full ^40^Ar/^39^Ar data are reported in Supplementary File #2.

## Detrital sanidine dating approach

The implemented sensitivity of the modern mass-spectrometers permits to date with great precision Pleistocene grains smaller than 400–300 μm. Combined with the continuous eruptive activity that characterized the volcanic region of central Italy during the last 800 ka^58^, and ref. therein, dating of sedimentary samples has become an extremely useful tool to assess the ages of aggradational successions deposited in response to sea-level rise during glacial terminations in the absence of intercalated, primary tephra layers (see Marra et al.^50^ for an in-depth discussion). In fact, when a statistically significant number of crystals is dated (i.e., 30–40 grains), it is reasonable to assume that the age of the youngest crystal population, besides providing a maximum age for the sedimentary deposit, should also be regarded as documenting the lack of crystals from younger eruptive products, implying that no younger eruptions occurred before the time of deposition. Such assumption allows to consider the youngest crystal age also an approximate minimum age (*terminus ante-quem*) (within the recurrence time of the volcanic activity) to the time of deposition of the sediment. As discussed in Marra et al.^50^ the youngest eruptions should be better represented in reworked, sedimentary deposits because their products crop out in wider areas than the older ones, which are buried under a longer sequence of strata. This consideration supports the principle that the age of a layer is bracketed between the ages of its youngest crystal population and of the next younger eruption, whose crystals do not occur in the layer but is documented in the area.

## Supplementary Information

Supplementary Figures.

Supplementary Tables.

## Data Availability

All data generated or analysed during this study are included in this published article [and its supplementary information files].
