# Supplementary material for: Terrestrial records of deglaciation events during terminations V and IV in the central Apennines (Italy) and insights on deglacial mechanisms
Source: Sci Rep. 2022 Nov 5;12:18770. doi: 10.1038/s41598-022-23391-7 (PMC9637136; doi:10.1038/s41598-022-23391-7)
Supplement: Supplementary file 1 — Supplementary Figures. [file 41598_2022_23391_MOESM1_ESM.pdf]

Terrestrial records of deglaciation events during terminations V and IV in the central Apennines (Italy) and insights on deglacial mechanisms

F. Marra[1]*, A. Pereira[2,3], B. Jicha[4], S. Nomade[5], I. Biddittu[6], F. Florindo[1], G. Muttoni[7,8], E.M. Niespolo[9,10], P.R. Renne[9,10], V. Scao[5]

[1] Istituto Nazionale di Geofisica e Vulcanologia, Rome, Italy
[2] Université Paris-Saclay, CNRS UMR 8148, GEOPS, France
[3] Département Hommes et environnements, Muséum national d'Histoire naturelle, Paris, France
[4]Department of Geoscience, University of Wisconsin-Madison, USA
[5] CEA Saclay, LSCE, UMR-8212, UVSQ-IPSL et Université Paris Saclay, Gif-sur-Yvette Cedex, France
[6] Istituto Italiano di Paleontologia Umana, Anagni, Italy
[7] Department of Earth Sciences, University of Milan, Milan, Italy
[8] ALP — Alpine Laboratory of Paleomagnetism, Peveragno (CN), Italy
[9] Department of Earth and Planetary Science, University of California, Berkeley, USA
[10] Berkeley Geochronology Center, Berkeley, USA

*Corresponding author: fabrizio.marra@ingv.it
ORCID ID 0000-0002-4881-9563

Supplementary Material #1 - Stratigraphic sections

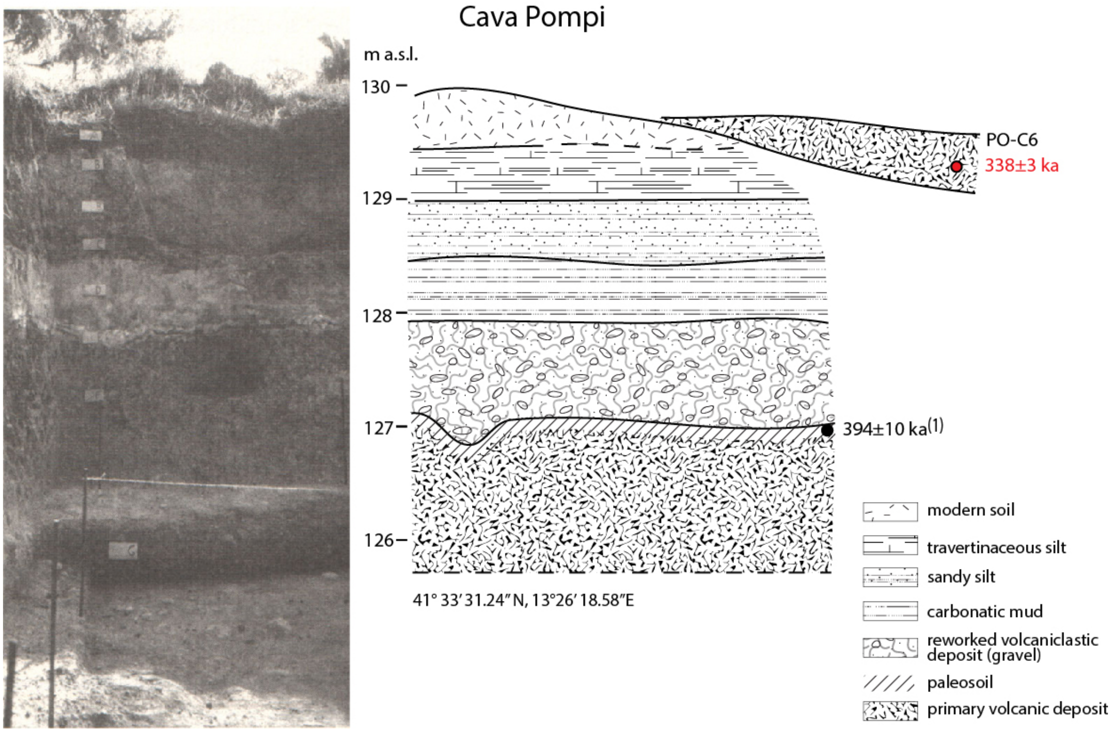

Figure S1 - Stratigraphic sketch of Cava Pompi archaeological section. Original photograph taken by author I. Biddittu. In red, $^{40}Ar/^{39}Ar$ age performed for the present study.

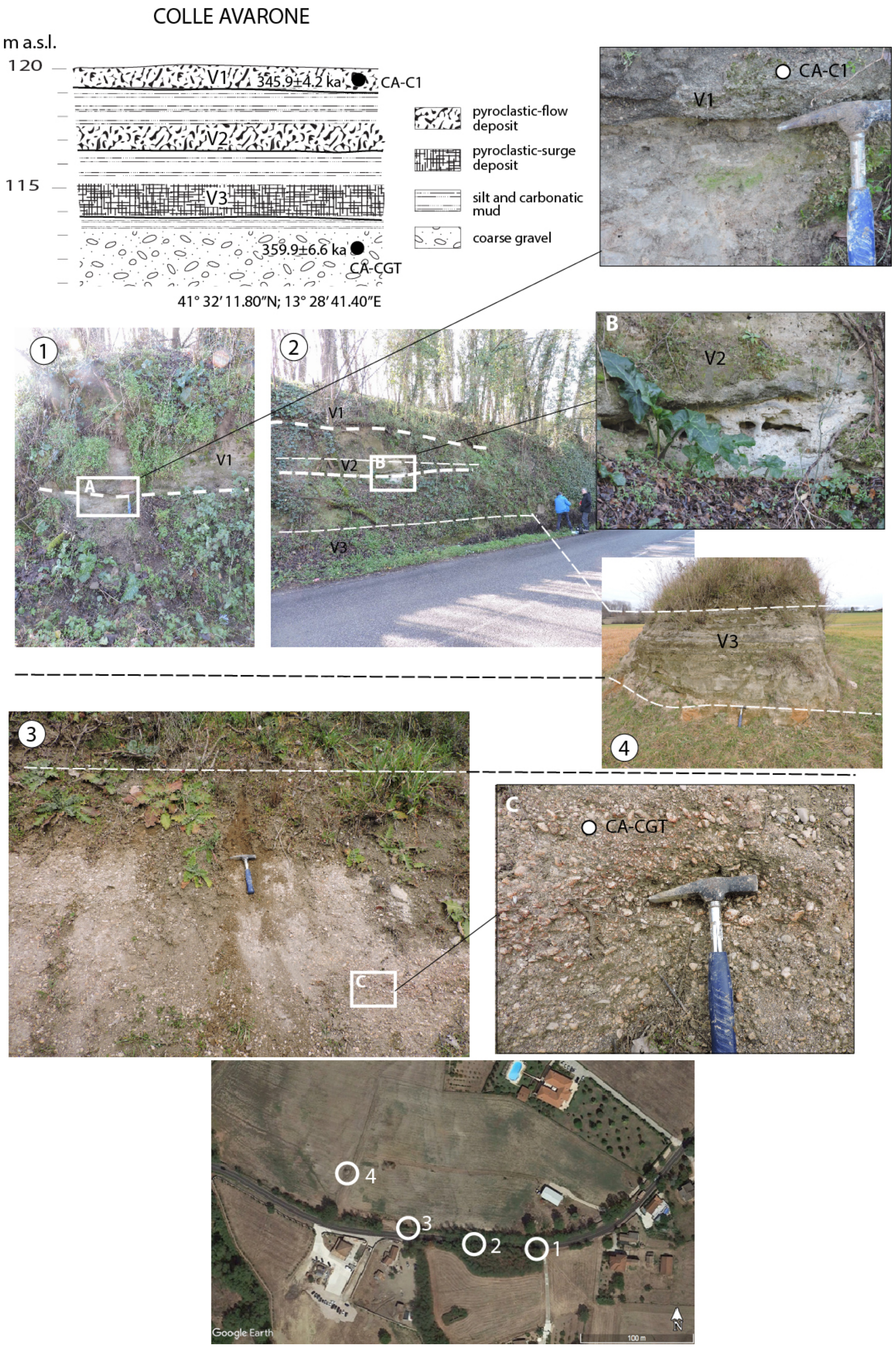

Figure S2 - Stratigraphic sketch of the composite Colle Avarone geological section. Photographs taken by author F. Marra.

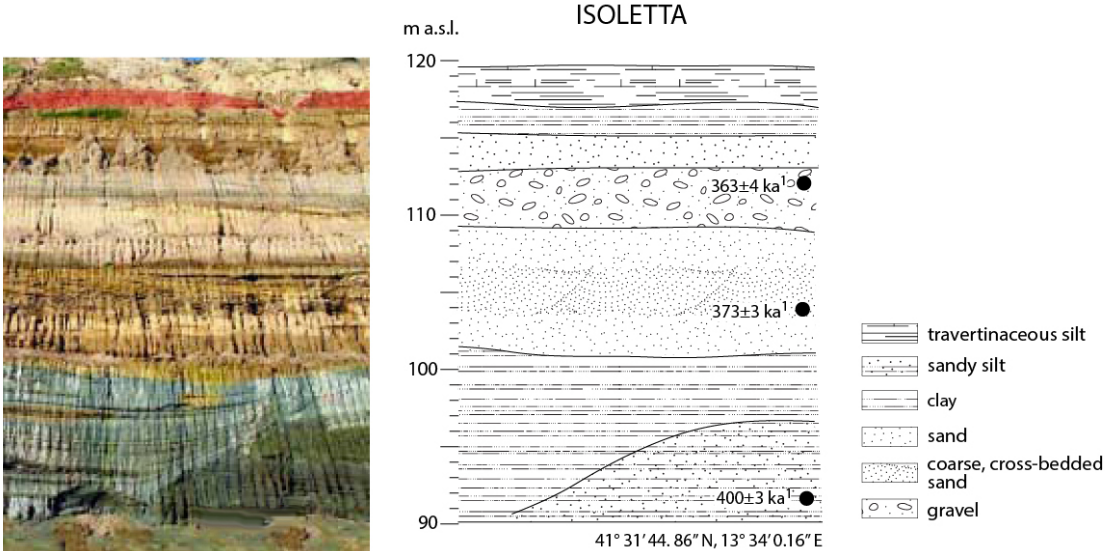

Figure S3 -Stratigraphic sketch of Isoletta geologic section. Photograph by author I. Biddittu.

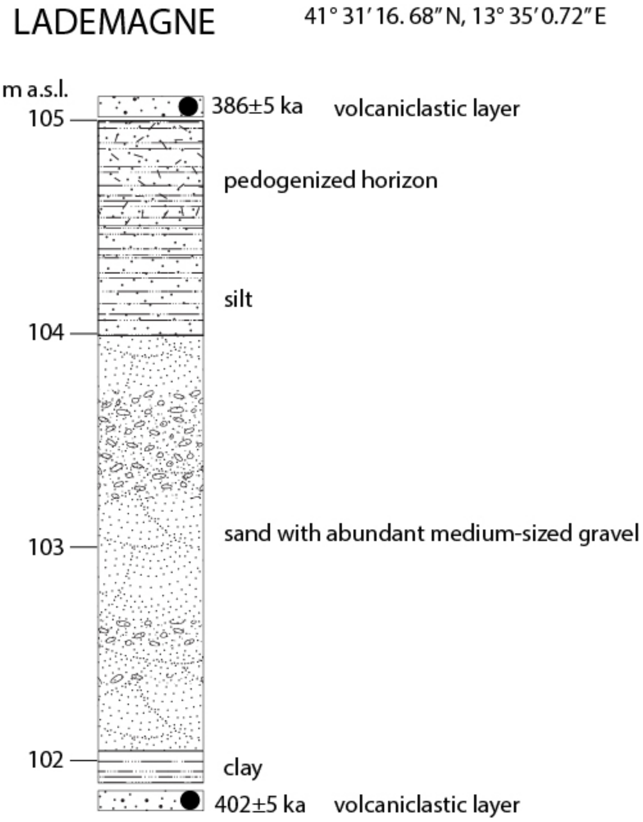

Figure S4 - Stratigraphic sketch of Lademagne geological section.

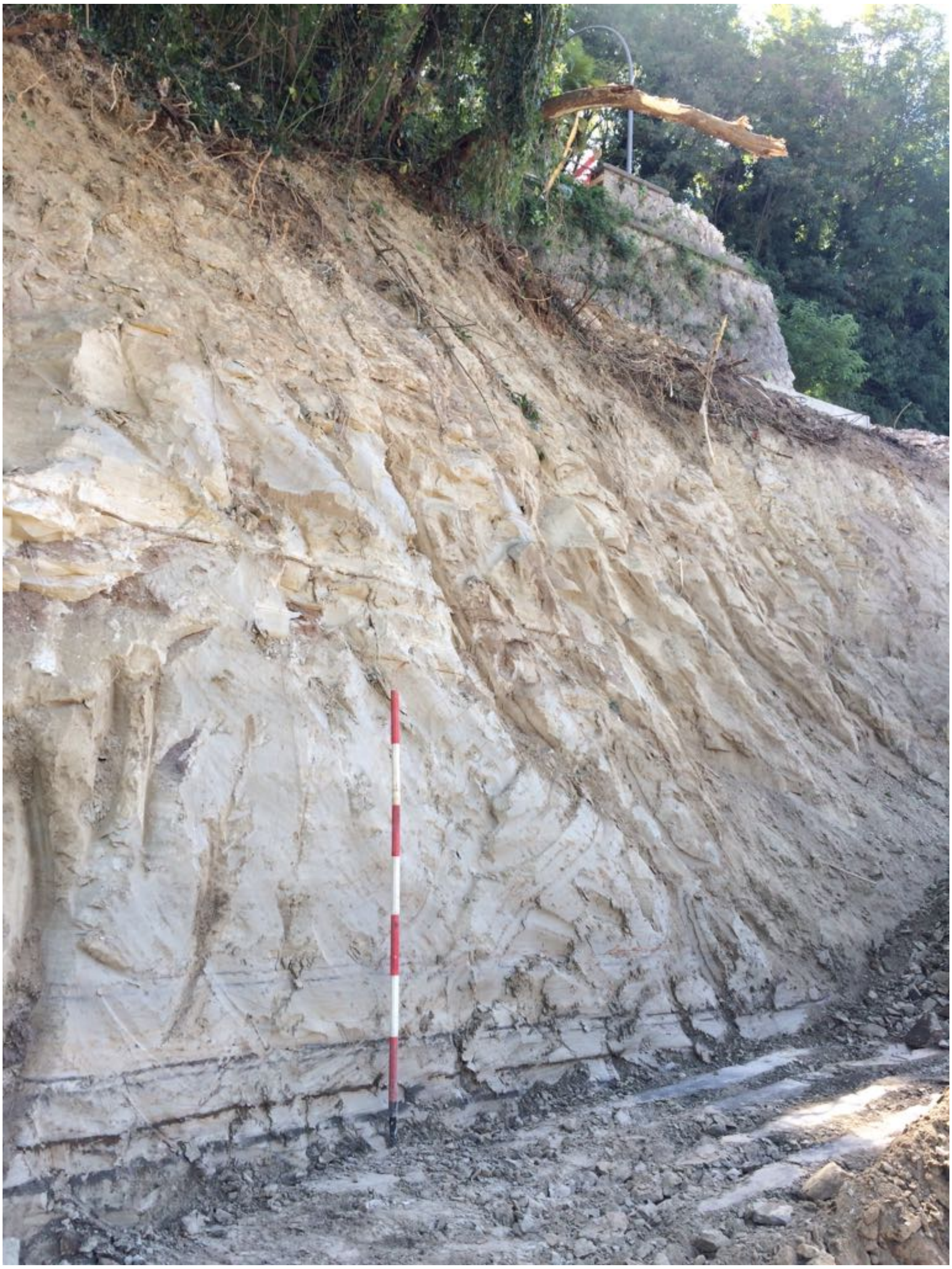

Figure S5 - Photograph taken by author I. Biddittu of the Pontecorvo outcrop showing occurrence of several tephra layers (arrow) intercalated in the Lower lacustrine succession. Two of these tephra layers were dated by K/Ar method[3].

REFERENCES

1. Pereira, A. *et al.* Geochronological evidences of a MIS 11 to MIS 10 age for several crucial Acheulian sites from the Frosinone province (Latium, Italy): Archaeological implications. *Quaternary Science Reviews* 187, 112-129 (2018).
2. Marra, F., Cardello, L., Gaeta, M., Jicha, B., Montone, P., Niespolo, E., Nomade, S., Palladino, D.M., Pereira, A., De Luca, G., Florindo, F., Frepoli, A., Renne, P., Sottili, G., 2021. The Volsci Volcanic Field (central Italy): an open window on continental subduction processes, International Journal of Earth Sciences 110:689–718. DOI:10.1007/s00531-021-01981-6
3. Narcisi. B, 1986. Ricerche di tefracronologia nella media e bassa Valle Latina. Mem Soc Geol It 35, 909–912.
